# Supplementary material for: Bacterial AB5 toxins inhibit the growth of gut bacteria by targeting ganglioside-like glycoconjugates
Source: Nat Commun. 2019 Mar 27;10:1390. doi: 10.1038/s41467-019-09362-z (PMC6437147; doi:10.1038/s41467-019-09362-z)
Supplement: Supplementary file 1 — Supplementary Information [file 41467_2019_9362_MOESM1_ESM.pdf]

Bacterial AB_5 toxins inhibit the growth of gut bacteria by targeting ganglioside-like glycoconjugates

Patry *et al.*

Supplementary Information

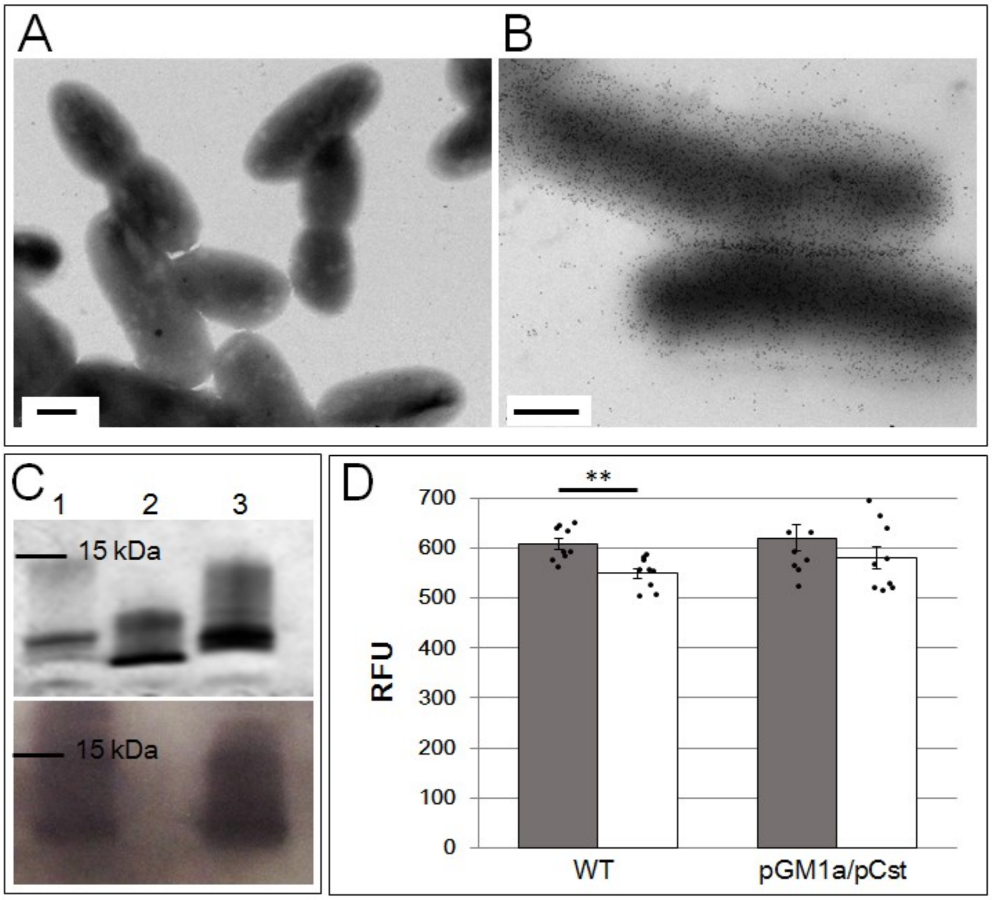

Supplementary Figure 1. Cholera toxin B subunit (CTB) binds to *Escherichia coli* engineered to express GM1 ganglioside-mimicking lipooligosaccharides (LOS) but does not impact cell permeability. Transmission electron microscopy following immunogold labeling with CTB was performed on **(A)** wildtype *E. coli* CWG 308, and **(B)** *E. coli* CWG 308 expressing pGM1/pCst (scale bars are 0.5 μm). **(C)** Upper panel: Silver stain of LOS isolated from cells shown in A (lane 1), cells shown in B (lane 2), and from LOS isolated from *C. jejuni* HS:19 (lane 3); lower panel: far western blot of the same samples using CTB as a probe. **(D)** Each *E. coli* strain was incubated with ethidium bromide for 20 min following treatment with PBS (white, n=9) or CTB (grey, n=9) and their relative fluorescence was measured. Error bars represent standard error of the mean (**p=0.0015, determined by two-tailed t-test). Source data are provided as a Source Data file.

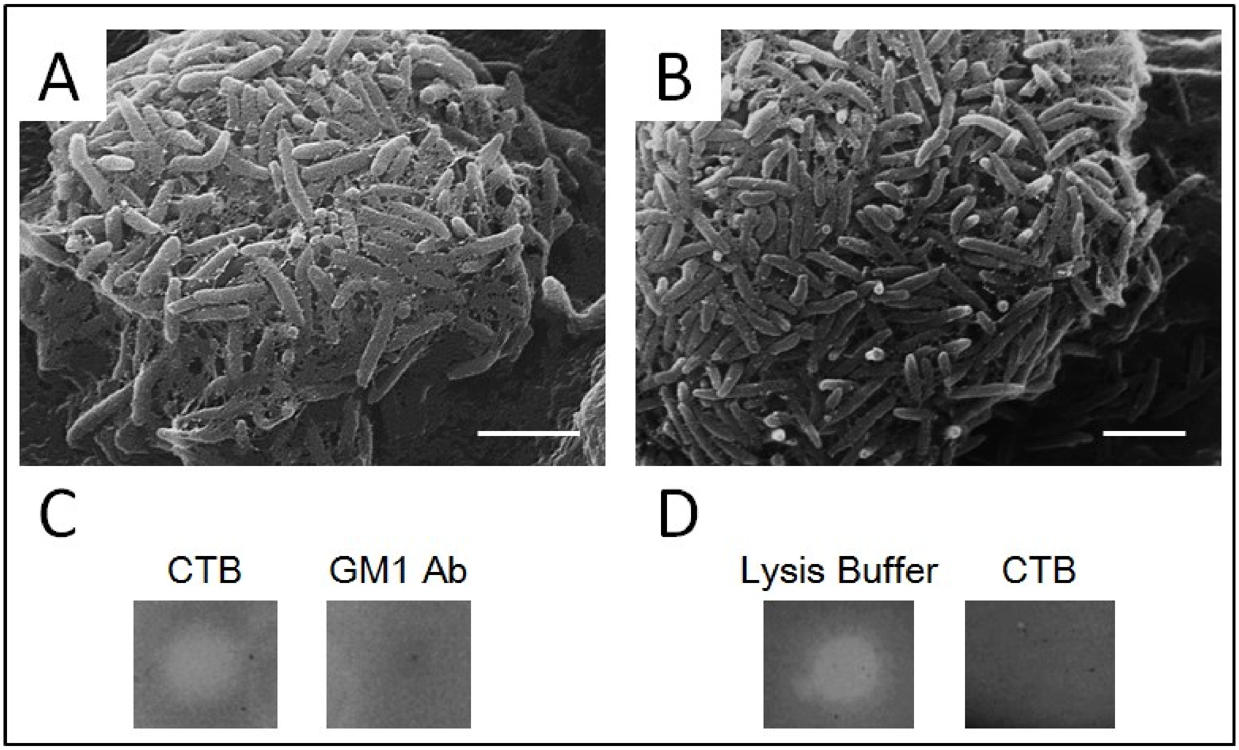

Supplementary Figure 2. Cholera toxin B subunit (CTB) is bacteriostatic and not bactericidal to *C. jejuni* HS:19. Scanning electron micrographs show surface morphology of *C. jejuni* HS:19 within **(A)** or outside **(B)** of the CTB zone of clearance. Agar clearance assays show the effect of various agents spotted either before **(C)** or after overnight *C. jejuni* HS:19 growth in soft agar **(D)**. Scale bars are 2 μm.

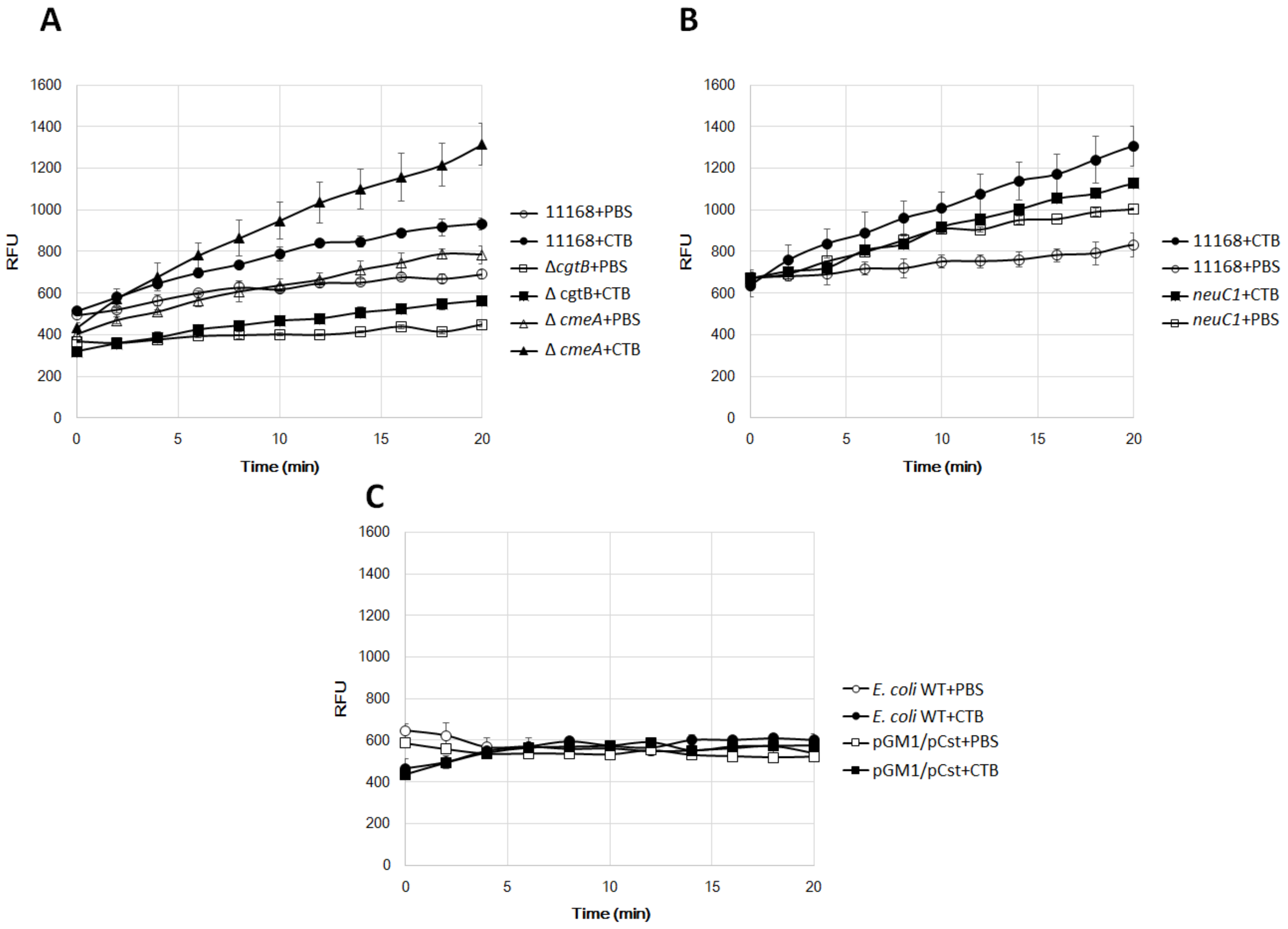

Supplementary Figure 3. Graphs from representative experiments showing the relative fluorescence of bacterial strains following treatment with ethidium bromide. (A) *C. jejuni* 11168 wildtype, Δ*cgtB* and Δ*cmeA,* **(B)** *C. jejuni* 11168 and *ΔneuC1,* or **(C)** *E. coli* CWG 308 and *E. coli* CWG 308 expressing pGM1/pCst were incubated with ethidium bromide for 20 min following treatment with PBS (control) or CTB and the relative fluorescence was measured (n=3 for each data point). Error bars represent standard deviation. Source data are provided as a Source Data file.

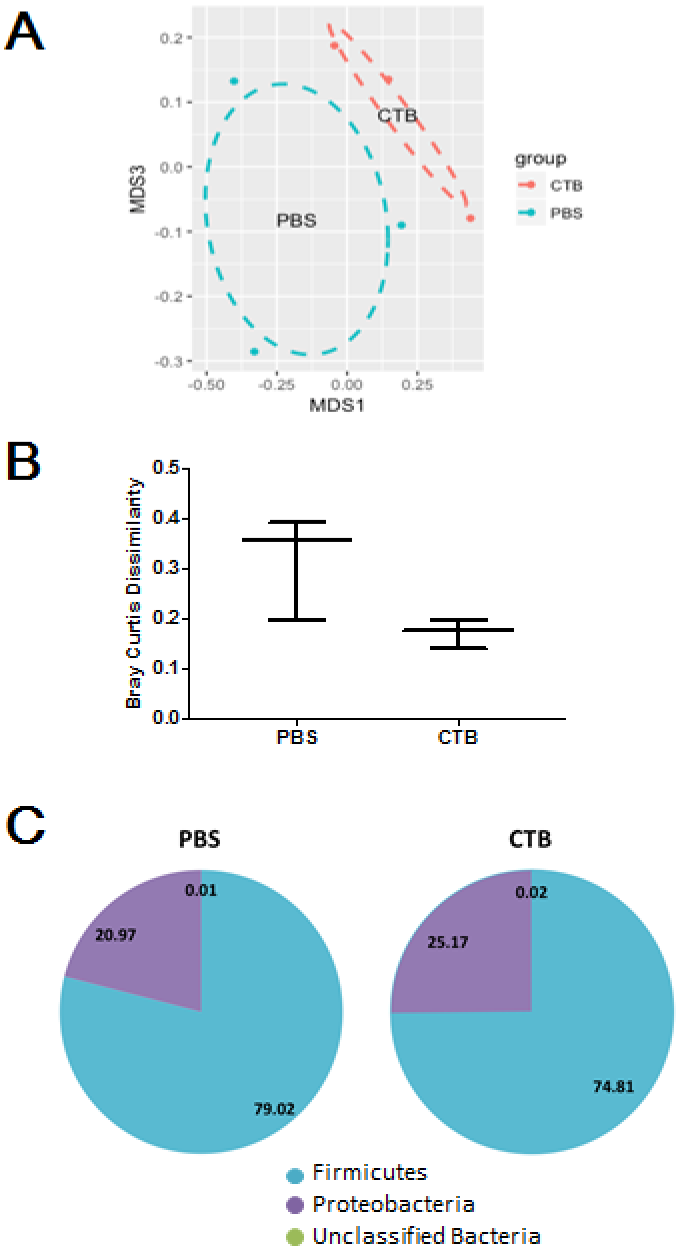

Supplementary Figure 4. Administration of CTB toxin to chickens results in shifts in the intestinal microbiome resembling trends observed with LTB. A small scale chicken experiment was done introducing either CTB (n=3) or PBS (n=3) and monitoring the relative changes in microbiome composition. **(A)** NMDS plot based on Bray Curtis distances show that introduction of CTB causes a shift in the gut microbiome of chickens relative to chickens treated with PBS. **(B)** Similar to the effect of LTB in the chicken gut microbiome, LTB decreases β-diversity without affecting α-diversity. **(C)** The toxin causes decreases in the relative abundance of Firmicutes with parallel increases in Proteobacteria. Statistical analyses: Adonis PERMANOVA test for A, Mann-Whitney test for B and C. Source data are provided as a Source Data file.

Supplementary Table 1. A list of primers used in this study.

Primer name	Sequence (5'-3')	Use
cgtB-mut-F-XhoI	ATATCTCGAGGAGCTAAAATGAGTCAAATT TCCATC	*C. jejuni* 11168 Δ*cgtB*
cgtB-mut-R-SacI	ATATGAGCTCGTTATGAAATTTTTAATATCT TTACGG	
InvPCR-cgtB-F-BamHI	ATATGGATCCGGACAATGTGGGCTAAAATA ATC	
InvPCR-cgtB-R-BamHI	ATATGGATCCGGATTTTTAGTTTTAAGTATT TGC	
neuC1F15	TTTTGTTAGCGGAACTAGAGCTGAT	*C. jejuni* 11168 *ΔneuC1*
neuC1R984	TAATACGACTCACTATAGGGATTTAAAATC GCTTCCAATATATCC	
CmeA-F	TTATTGTGCTCCAATTTCTTTAACTTC	*C. jejuni* 11168 *ΔcmeA*
CmeA-R	ATGAAATTATTTCAAAAAAATACTATTTTAG	
CmeA-os-R	TAGGAGGGGTTAAAGAAGGATATTG	
CmeA-os-F	TTTCTAAATGGAATCAATAGCT	
DL39	TTAAGAGCAAGATATGAAGGTG	*cgtB* gene sequencing
DL41	CCATTTGAATTGATATTTTTG	
primer 1139R	TTAGCCCACATTGTCCAAAA	
926F	AAACTYAAAKGAATWGRCGG	16S rRNA sequence analysis
1392R	ACGGGCGGTGWGTRC	
926R	ACCGCTTGTGCGGGCCC	
515F	GTGCCAGCMGCCGCGGTAA	
1068F	GCATGGCYGYCGTCAG	
1391R	GACGGGCGGTGTGTRCA	
519R	GWATTACCGCGGCKGCTG	
27F	AGAGTTTGATCCTGGCTCAG	
1492R	GGTTACCTTGTTACGACTT	

R=A or G, Y=C or T, W=A or T, K=G or T, M=A or C

Supplementary Table 2. List of BioSample accession numbers for the 16S rRNA datasets.

Sample name	Accession number	Sample name	Accession number
454	SAMN10962775	467	SAMN10962788
455	SAMN10962776	468	SAMN10962789
456	SAMN10962777	469	SAMN10962790
457	SAMN10962778	470	SAMN10962791
458	SAMN10962779	471	SAMN10962792
459	SAMN10962780	472	SAMN10962793
460	SAMN10962781	301	SAMN10962794
461	SAMN10962782	302	SAMN10962795
462	SAMN10962783	303	SAMN10962796
463	SAMN10962784	304	SAMN10962797
464	SAMN10962785	305	SAMN10962798
465	SAMN10962786	306	SAMN10962799
466	SAMN10962787		
